# Single-Cellular Biological Effects of Cholesterol-Catabolic Bile Acid-Based Nano/Micro Capsules as Anti-Inflammatory Cell Protective Systems

**DOI:** 10.3390/biom12010073

**Published:** 2022-01-04

**Authors:** Armin Mooranian, Corina Mihaela Ionescu, Daniel Walker, Melissa Jones, Susbin Raj Wagle, Bozica Kovacevic, Jacqueline Chester, Thomas Foster, Edan Johnston, Jafri Kuthubutheen, Daniel Brown, Marcus D. Atlas, Momir Mikov, Hani Al-Salami

**Affiliations:** 1 The Biotechnology and Drug Development Research Laboratory, Curtin Medical School & Curtin Health Innovation Research Institute, Curtin University, Bentley, Perth, WA 6102, Australia; A.Mooranian@curtin.edu.au (A.M.); c.ionescu@postgrad.curtin.edu.au (C.M.I.); daniel.walker1@postgrad.curtin.edu.au (D.W.); melissa.a.jones@postgrad.curtin.edu.au (M.J.); susbinraj.wagle@postgrad.curtin.edu.au (S.R.W.); bozica.kovacevic@postgrad.curtin.edu.au (B.K.); j.chester@student.curtin.edu.au (J.C.); thomas.p.foster@student.curtin.edu.au (T.F.); edan.johnston@student.curtin.edu.au (E.J.); 2 Hearing Therapeutics, Ear Science Institute Australia, Queen Elizabeth II Medical Centre, Nedlands, Perth, WA 6009, Australia; marcus.atlas@earscience.org.au; 3 Fiona Stanley Hospital, Murdoch, WA 6150, Australia; Jafri.Kuthubutheen@health.wa.gov.au; 4 Curtin Medical School & Curtin Health Innovation Research Institute, Curtin University, Bentley, Perth, WA 6102, Australia; daniel.brown2@curtin.edu.au; 5 Department of Pharmacology, Toxicology and Clinical Pharmacology, Faculty of Medicine, University of Novi Sad, 21101 Novi Sad, Serbia; momir.mikov@mf.uns.ac.rs

**Keywords:** chenodeoxycholic acid, microencapsulation, inflammation, interleukins, cytokines

## Abstract

Recent studies in our laboratories have shown promising effects of bile acids in ➀ drug encapsulation for oral targeted delivery (via capsule stabilization) particularly when encapsulated with Eudragit NM30D^®^ and ➁ viable-cell encapsulation and delivery (via supporting cell viability and biological activities, postencapsulation). Accordingly, this study aimed to investigate applications of bile acid-Eudragit NM30D^®^ capsules in viable-cell encapsulation ready for delivery. Mouse-cloned pancreatic β-cell line was cultured and cells encapsulated using bile acid-Eudragit NM30D^®^ capsules, and capsules’ images, viability, inflammation, and bioenergetics of encapsulated cells assessed. The capsules’ thermal and chemical stability assays were also assessed to ascertain an association between capsules’ stability and cellular biological activities. Bile acid-Eudragit NM30D^®^ capsules showed improved cell viability (e.g., F1 < F2 & F8; *p* < 0.05), insulin, inflammatory profile, and bioenergetics as well as thermal and chemical stability, compared with control. These effects were formulation-dependent and suggest, overall, that changes in ratios of bile acids to Eudragit NM30D^®^ can change the microenvironment of the capsules and subsequent cellular biological activities.

## 1. Introduction

Pharmaceutical compound delivery, that is, the development of drug delivery systems (DDS) is a highly significant and, accordingly, an extensively researched discipline [1], where DDS optimization is attained by maximization of medicinal impact and minimization of side effects [2]. M-based vehicles for local drug delivery have become an area of increasing interest and relevance due to the potential for therapeutic results to be attained without unwanted secondary effects associated with systemic delivery [3]. Nanoparticles provide valuable applications in the field of encapsulated cell delivery, such as for the transplantation of viable pancreatic β-cells for sustained insulin release in Type 1 diabetes (TID) patients [4].

Chenodeoxycholic acid (CDCA) is an endogenous primary bile acid (BA), which has been found to improve permeation of poorly soluble drugs [5]. The rationale of bile acid inclusion is due to their pharmaceutical and pharmacological as well as promising endocrinological effects in cell encapsulation and biological performance, post encapsulation [6]. Recent studies by our laboratory have demonstrated that when the BA CDCA is added to alginate and used for microencapsulation of pancreatic β-cells, the β-cells displayed improved cellular viability. Furthermore, microcapsules containing CDCA displayed improved structural integrity and induced an attenuated inflammatory response, implying auspicious future perspectives for the use of BAs in β-cell transplantation [7]. Additionally, our group revealed that when incorporating a highly lipophilic BA (LBPA) to enhance cellular viability of microencapsulated β-cells, specific microencapsulating parameters modified capsule properties, and through this we discovered optimal capsule formulation methodology [8]. Lastly, we recently performed analysis to reveal the effect of BA’s addition on the electric charge of microcapsules. Ursodeoxycholic acid (UDCA) was added to a novel formulation containing high ratios of poly-L-ornithine (PLO) and suspended in electrical-stimulation hydrogel and polystyrene sulphone. Changes in electric charge indicated that BA addition augmented both cell viability and function [9]. These experiments highlight the positive impact of BAs on microcapsule characteristics. Further improvements can still be made, however, through optimization of both formulation research and encapsulation methodology. One of the polymers that has shown promising potential is Eudragit. 

Basnet et al. investigated the viability of Eudragit RS 100 (ERS-100) and Eudragit RL 100 (ERL-100) as microencapsulating polymers, with particular focus on stability parameters through time. Two sets of microcapsules were analyzed: one new batch, and one that had been stored at room temperature for ten years. Structural analysis of microcapsules revealed a lack of interaction between ERS-100/ERL-100 and core material in the sample stored for ten years, reflecting its stability [10]. Eudragit S100-PLGA (poly (lactic-co-glycolic)) nanocapsules were used as vehicle for the delivery of a time-sensitive anti-cancer drug in a recent study performed by Pandey et al. Nanocapsules with active drug were fully absorbed into the HT 29 adenocarcinoma cells, reflecting the effectiveness of the optimized Eudragit nanoparticles as a transport mechanism for improved colon targeting [11]. A study conducted by Kim and Ki-Seok explored the impact of ERS-100-based microcapsules’ drug release profiles. Although encapsulation efficiency and drug loading were impaired, microcapsules containing ERS-100 exhibited an accelerated drug release rate [12]. In another study by Azarmi et al., Eudragit RL/RS matrices were exposed to varying thermal intensities and then tested for tensile strength and drug release rates. Eudragit tablets exposed to 40°C thermal treating were unaffected and exhibited normal drug release rates, indicating Eudragit remains functional within the internal environment of the human body. However, increased duration of thermal treating negatively impacted drug release, and so should also be considered [13]. 

Further analysis of Eudragit by Broughton and Sefton examined its impact when incorporated in the encapsulation of Chinese hamster ovary fibroblasts (CHOFs) cells. An inverse relationship between growth of CHOF cells and capsule quality was discovered, indicating that Eudragit should not be considered for the encapsulation of pancreatic islet cells [14]. However, Douglas and Sefton from the Department of Chemical Engineering and Applied Chemistry at the University of Toronto further explored permeability of microencapsulated pancreatic islet cells, finding contrary results. A Thiele modulus/Biot number analysis of glucose expenditure indicated only a small effect on glucose diffusion in islet cells, which was consistent with experimental results. Therefore, it was concluded that the Eudragit coating does not attenuate glucose diffusion, and is a viable option for the encapsulation of pancreatic islet cells [15]. 

Accordingly, the current study aimed to examine the BA-Eudragit NM30D^®^ augmentation of capsules containing viable mouse cloned Pancreatic β-cell (Min-6) populations ready for delivery. Thus, the impact of the CDCA-Eudragit NM30D^®^-based formulations on encapsulation, cell performance, and drug delivery were examined by various methods. These included, drug release profiles, microcapsule imaging, inflammatory biomarkers, various single-cell bioenergetics tests, and chemical and physical stability analyses.

## 2. Materials and Methods

### 2.1. Materials

SA (low viscosity sodium alginate, 99%), PLL, PVP, PVA, and CDCA were purchased from Sigma Chemical Co (St. Louis, MO, USA) and Eudragit form Evonik Industries (Essen, Germany). Calcium chloride dehydrate (CaCl_2_.2H_2_O, 98%) was obtained from Scharlab S.L., Australia. 

### 2.2. Cell Culture and Encapsulation 

Min-6 cells were cultured under sterile conditions as per our standard laboratory procedures [7,16,17]. Dulbecco’s Modified Eagle’s Medium (DMEM) media from Thermo fisher Scientific^®^ (Waltham, MA, USA) was combined with antibiotics and 10% fetal bovine serum in T-25 cm^2^ flasks, all of which were also purchased from Thermo Fisher Scientific^®^ (Sydney, NSW, Australia). Encapsulated cells were formed with varying excipients as described below. All formulations contained 1.6% sodium alginate, 2% Eudragit NM30D^®^, 1.5% poly-L-lysine (PLL), polyvinylpyrrolidone (PVP) and polyvinyl acetate (PVA) as well as concentrations (%) of CDCA of 0.3, 0.5, 0.8, 1.1, 1.3, 1.5, 1.8 and 3, with (%) of poloxamer ranging from 3 to 11. Encapsulation of cells was consistent with our previous methods [6,18], utilizing a Büchi Ionic Gelation Vibrational Jet Flow technology system and a 2% CaCl_2_ bath.

### 2.3. Microcapsule Imaging 

Freshly encapsulated cells were mounted onto glass slides for observation under light microscope, utilizing a calibrated scale and 4× objective lens. The light microscope was attained from Olympus Company (Tokyo, Japan), model: Olympus IX-51. Capsule topographical analysis was also carried out via scanning electron microscopy (SEM). Capsules were clad in a 5 nm platinum coat and placed in a vacuum for imaging. Images were generated using 2.5-nm calibrated resolution at 3 kV, with a MIRA3 field emission SEM (Tescan, Brno, Czech Republic). Finally, in-house developed confocal imaging techniques involving fluorescent biomarkers were used to generate images depicting distribution of viable cells within capsules [7,8]. Min-6 cell staining was undergone with carboxyfluorescein succinimidyl ester (CFSE) from a cell proliferation kit (Life Technologies, Carlsbad, CA, USA) and subsequently analyzed using UltraVIEW Vox spinning disk confocal microscope (Perkin Elmer, Waltham, MA, USA) [4]. 

### 2.4. Cell Viability and Insulin Production Analyses

Cellular viability was assessed using MTT (3-[4,5-dimethylthiazol-2-yl]-2,5 diphenyl tetrazolium bromide) assay as per our established methods [4,19]. MTT stock solution from Sigma Chemical CO (St. Louis, MO, USA) was added to buffer, which was then adjusted to the appropriate pH of 7.4. Following this, sterile filtration was used to remove undissolved particles, 20 µL aliquots of MTT stock were then added to wells of a 96 well plate (Thermo fisher Scientific, Waltham, MA, USA). Encapsulated cells were added to wells and subsequently incubated for 48 h before cell viability by colorimetric assay was determined. Insulin secretion by cells was quantified using a highly sensitive Mouse Insulin ELISA (Mercodia Cooperation, Uppsala, Sweden) [20,21]. In the interest of validity, insulin production levels of formulations were normalized dependent upon the cell count of that formulation. 

### 2.5. Inflammatory Profile Analyses

Inflammatory profiles on encapsulated cells were assessed using a BD Biosciences (San Jose, CA, USA) cytokine bead array (CBA) system in conjunction with an Attune Acoustic Focusing Flow Cytometer, from Life Technologies (Carlsbad, CA, USA) [4,8,20]. Various pro-inflammatory cytokine levels were determined, including interferon-gamma (IFN-gamma), interleukin1-beta (IL1-beta), and tumour necrosis factor-alpha (TNF-alpha), as well as the anti-inflammatory interleukin-10 (IL-10). Again, in order to maintain validity, biomarker quantities were normalized based upon the number of viable cells within its associated formulation.

### 2.6. Cell Biological Activity Analyses

Encapsulated cell health was determined by mitochondrial activity using a Seahorse Flux Analyser XF 96 from Seahorse Bioscience (Santa Clara, CA, USA) in a manner consistent with those developed by our laboratory [7,19]. In real-time per-minute exposure, alterations in proton and oxygen levels were highlighted by fluorescence of biosensors [22,23], followed by repeated injections using a multichannel dispenser to determine mitochondrial respiration and associated parameters [4].

### 2.7. Chemical and Thermal Analyse

Chemical examination of encapsulated cell formulations was determined via Fourier transform infrared analysis (FTIR). This spectral analysis was performed using the FTIR spectrometer-TWO, from PerkinElmer Inc. (Waltham, MA, USA) with a pre-set frequency range of 450–4000 cm^−1^. This allowed the determination of chemical atomic bond transmittance of formulation excipients combined and individually, and also of the formed micro capsules. A PerkinElmer DSC 8000 (PerkinElmer Inc. Waltham, MA, USA) was utilized for a differential scanning calorimetric (DSC) analysis to determine thermal parameters. After placement within a sealed aluminum dish a nitrogen flow rate of 30 mL/ minute was used to raise sample temperature by 30 °C/min [24].

### 2.8. Statistical Analysis and Graphing 

Statistical analysis was done on triplicates of data (*n* = 3) with mean data displayed graphically. GraphPad Prism software (San Diego, CA, USA) was used to generate all graphs and complete statistical tests, in the latest version (version 9.1.0). Analysis was undergone by ordinary one way ANOVA’s using a significance level of *p* < 0.05. Formulae where 0.01 < *p* < 0.05 are denoted as having one asterisk (*), whereas a double asterisk (**) denotes those formulae for which *p* < 0.01. 

## 3. Results and Discussion

### 3.1. Capsule Topographical Analysis and Morphology 

**Figure 1:**
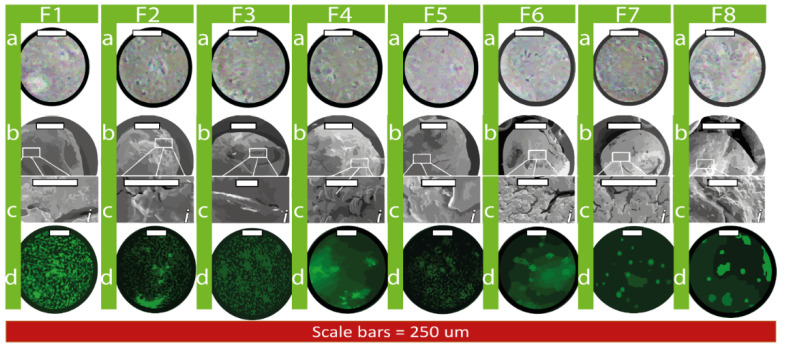
Surface morphology by light microscope (**a**), SEM (**b**,**c**), and distribution of CFSE-stained cells via confocal microscopy (**d**).

**Figure 2:**
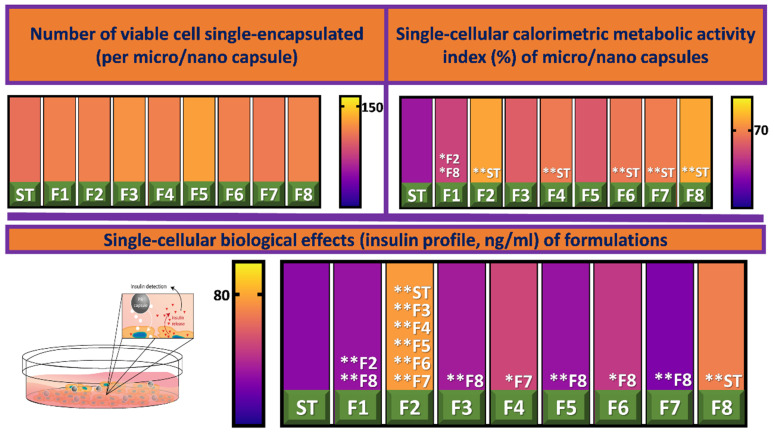
Cell viability (**top**, **left**), metabolic activity (**top**, **right**), schematic representation of experimental theory (**middle**), and insulin profile (**bottom**). Data are mean ± standard error of the mean, *n* = 3. Singular asterisk denotes where *p* < 0.05, whereas double asterisk denotes *p* < 0.01.

Size distribution and polydispersity index as well as zeta potential showed consistent results with our published studies [6,19,20,25]. Light microscopy (a) of encapsulated cells is displayed in Figure 1 below. In accordance with the optical reflectional theory, all formulations appear to depict viable cell populations when viewed under light microscope [26]. Capsule shape (b) and fine topographic detail (c) is shown by means of SEM micrographs. Overarchingly, all capsules conjugated with Eudragit NM30D^®^ (F1–F8) display consistent spherical/ ovoid shape, with minor topographical variation. Brightness of CFSE stained cells under confocal microscopy (d) is subject to cell distance from micro/nano capsule surface. As seen in Figure 1, the internal complexity and external structure of cells are similar among the formulations, which indicates less significant impact of bile acid on the formulation; however, the effect of BA can be clearly seen on cell’s number and viability, which is presented in Figure 2. All formulations depict robust encapsulation of viable cells, but further analysis by means of MTT assay is required for quantification of viable cell populations. 

### 3.2. Cell Viability and Insulin Production

As displayed in Figure 2 below, all formulations, one to eight, that contained Eudragit NM30D^®^ and CDCA (at varying concentrations) exhibited high levels of viable cell populations. Though, as did the Sham Trial (ST); and no statistically significant distinctions in cell populations were observed between any formulations. However, when observing single-cell calorimetric metabolic activity formulations: two, four, and six to eight display significantly higher levels of metabolic activity (*p* < 0.01), indicating increased function. Furthermore, levels of insulin production were found to be significantly higher than ST in formulations two and eight (*p* < 0.01). Accordingly, it appears that CDCA-Eudragit NM30D^®^ encapsulated Min-6 cells functionality is augmented, specifically at excipient ratios depicted by F2 and F8. This has been schematically represented in Figure 2. CDCA’s enhancing effect on insulin production has been highlighted in our earlier work; it was hypothesized that this may be a result of direct interaction between CDCA and β-cells at the molecular level [7]. BAs such as CDCA are known to act upon the Farnesoid-X-Receptor (FXR), which is expressed in pancreatic β-cells. Animal studies have revealed that activation results in increased insulin secretion, improved blood glucose control, and reducing harmful manifestations associated with diabetes [27,28]. These findings contradict those of earlier work by our laboratory, whereby the inclusion of a tertiary BA decreased insulin production and viability of pancreatic β-cells [29]. However, it should be noted that different excipients were involved in that study, and BAs’ beneficial effect on biological processes of β-cells has been displayed numerous times by our laboratory [7,16,17,19,30,31]. These incongruencies, along with the inter-formulation differences in therapeutic effects of the current study, support the notion that BAs exert therapeutic or deleterious effects based upon the ratios in which they are used, and the scope of the pharmacological context at hand. Further light is shed on this topic by analysis of the immunogenic/ inflammatory consequences brought about by BA addition.

### 3.3. Inflammatory Profile

**Figure 3:**
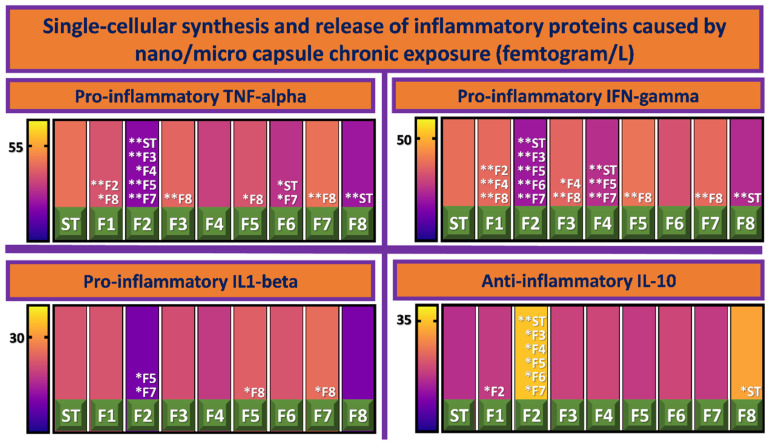
Single-cellular synthesis and release of inflammatory proteins caused by nano/micro capsule chronic exposure (femtogram/L). Inflammatory proteins include TNF-alpha (**top**, **left**), IFN-gamma (**top**, **right**), IL1-beta (**bottom**, **left**), and IL-10 (**bottom**, **right**). Data are mean ± standard error of the mean, *n* = 3. Singular asterisk denotes where *p* < 0.05, whereas double asterisk denotes *p* < 0.01.

One of the major limiting factors for the practicality of live-tissue transplant for the treatment of disease is the risk of immune response and subsequent injury; which is why pharmaco-immunologic therapies form a fundamental part of organ failure management [32]. Post-transplant care of patients receiving islet transplants requires immunosuppressive therapy; however, such therapy generates deleterious and malignant side effects, such as infection and organ toxicity [33]. Thus, micro/ nano capsule formulations, which function as immune-barriers, pose great potential in islet transplantation; [21] highlighting the relevance of formulation inflammatory profile analysis. As seen in Figure 3, the entire cohort of CDCA-Eudragit NM30D^®^ formulations’ levels of pro-inflammatory cytokines (TNF-alpha, IFN-gamma, and IL1-beta) were relatively low. F2 and F8 showed the most promising inflammatory profiles, both generating significantly lower levels of TNF-alpha and IFN-gamma (*p* < 0.01), and significantly higher levels of the protective anti-inflammatory IL-10 (*p* < 0.01 and *p* < 0.05 respectively), when compared to ST. We have previously detailed the role of polymers, hydrogels, and polyelectrolytes on cell viability and functionality [34]. The comparison between F2 and F8 is to illustrate the role of bile acid CDCA in influencing cell functionality, given that the other ingredients have previously been shown to not influence cell-based assays. Diabetes-related damage to pancreatic β-cells is associated with an imbalance of inflammatory cytokines, whereby pro-inflammatory cytokines outweigh the protective effect of anti-inflammatory cytokines. Elevated levels of TNF-alpha, IL1-beta, and IFN-gamma are observed in both the pancreatic islets and blood of diabetic patients. When pro-inflammatory cytokines accumulate within β-cells, they activate metabolic pathways leading to cell death by apoptosis. Specifically, IFN-gamma and IL1-beta trigger apoptotic pathways using transcription factors STAT-1 and nuclear factor kappa light-chain-enhancer of activated B cells (NF-κB) [35]. IL-10 is the strongest anti-inflammatory cytokine, possessing the ability to decrease TNF-alpha, IFN-gamma, and IL1-beta secretion from activated macrophages. Furthermore, IL-10 down-regulates pro-inflammatory cytokine receptors and up-regulates anti-inflammatory cytokine receptors, counteracting inflammatory processes and therefore associated damage [36].

### 3.4. Cellular Bioenergetics

**Figure 4:**
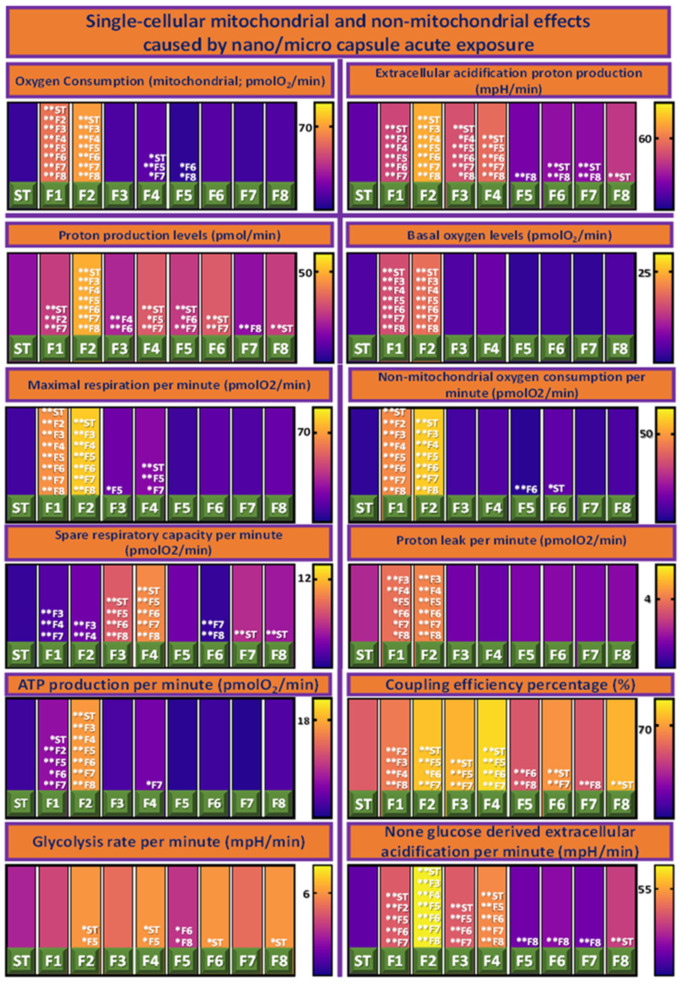
Various single-cellular mitochondrial and non-mitochondrial effects caused by micro/nano capsule acute exposure. Data are mean ± standard error of the mean, *n* = 3. Singular asterisk denotes where *p* < 0.05, whereas double asterisk denotes *p* < 0.01.

The pathogenesis of diabetes is associated with mitochondrial dysfunction, as mitochondrial metabolism is a prime regulator of insulin secretion [37]. Accordingly, various mitochondrial and non-mitochondrial bioenergetics have been assessed—as observed in Figure 4. F1 and F2 generated significantly higher (*p* < 0.01) levels of basal oxygen and oxygen consumption than the ST, implying increased mitochondrial respiration [38,39]. Compounding this, these formulations displayed increased levels of both maximal respiration (per minute) and non-mitochondrial oxygen consumption, which were both significant against ST (*p* < 0.01). Oxygen molecules function as electron carriers in the electron transport chain (ETC), thus the enhanced respiration may be linked with the facilitation of ATP production and subsequent insulin secretion [19,40]. Formulations 3–8 exhibited substantially lower basal oxygen levels, oxygen consumption, maximal respiration, and non-mitochondrial oxygen consumption. Such inter-formulation differences imply that the variation in constituents of these formulations altered the cellular microenvironment in some way to cause altered oxygen perfusion and/ or use. This is a highly relevant finding in light of the fact that a major challenge in the transplantation of islet cells is risk of oxygen deficiency [41]. It should be noted that F1 and F2 possess a significantly lower spare respiratory capacity when compared to F3 and F4 (*p* < 0.01)—which were the highest in this class. 

Proton leak refers to the return of protons to the mitochondrial matrix independent of ATP synthase, resulting in the uncoupling of substrate oxidation and ATP synthesis [42]. Protons are able to move in this way due to the presence of uncoupling proteins (UCP’s) on the inner mitochondrial membrane; UCP-2 is prevalent in the pancreatic islets and is a down-regulator of insulin secretion [43,44]. Although F2 displayed significantly higher levels of proton leak than F3–F8 (*p* < 0.01), it was not found to be significantly higher than ST. Additionally, F2 was found to have significantly higher extracellular acidification rate and levels of proton production compared to all other formulations and the ST (*p* < 0.01). This raises the possibility that this increased proton leak is secondary as a result of the increased proton concentration within F2 cells, in accordance with Flicks Law of Diffusion. Coupling efficiency (CE) depicts the proportion of respiration from islet cells, which is used for ATP production [37]. The CE of F2–F4, F6, and F8 were found to be significantly higher than the ST (*p* < 0.01), suggesting that encapsulation of islet cells augmented mitochondrial efficiency and thus functionality.

Levels of ATP production of F2 were far higher than that of all other formulations including the ST (*p* < 0.01). This result is consistent with expectations, considering this formulation’s oxygen consumption, CE, and insulin profile, as previously discussed. Higher levels of ATP production are likely a function of the increased amount of oxygen molecules functioning as electron receivers in the ETC amplifying the propensity of oxidative phosphorylation [23,40,45]. In the mitochondria, pyruvate is oxidized to NADH generating a proton gradient; protons are then utilized by ATP synthase to convert ADP into ATP, altering the ATP:ADP ratio [46]. This ratio is consequential to pancreatic β-cells as they are stimulated to secrete insulin when it is low, which eventuates in increased ATP levels, as has been observed here [45]. Moreover, F2 along with F4 and F8 lead the cohort in glycolysis rate, likely increasing cellular energy production and CE. It proves likely that these results lead to far greater levels of non-glucose derived extracellular acidification, which was seen in F2 (*p* < 0.01). When encapsulated, cellular biological function is likely to be affected by the chemical and thermal properties of the structure encapsulating them; accordingly, these features require analysis.

### 3.5. Chemical and Thermal Analyses

**Figure 5:**
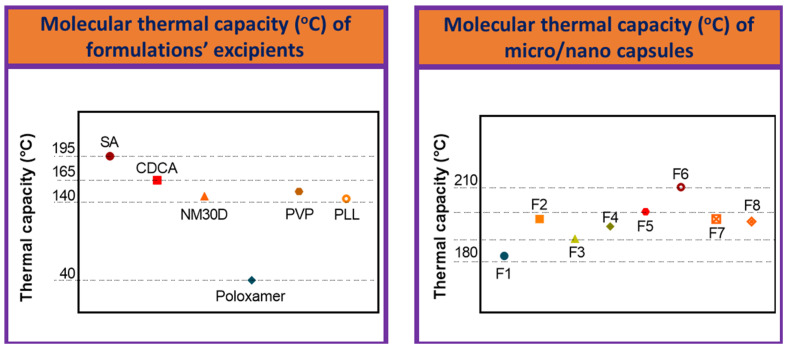
Molecular thermal capacity (°C) of formulation excipients (**left**) and micro/nano capsules (**right**). Data are mean ± standard error of the mean, *n* = 3.

The percentage transmittance (%T) of the primary chemical bond of formulation excipients is displayed in Table 1. Eudragit NM30D^®^ shows a value exceeding 300 %T, while CDCA and PLL Primary chemical bond transmittance were also among the highest, of these constituents. For this, two major peaks of %T were observed, which represent the two chemical structures seen most commonly on micro/nano capsules of that formulation. The significant differences in %T between excipients (which were generally higher) and formulations (which were generally lower, for both peaks) suggest stable levels of chemical bond formation on the molecular level [47]. Our previous study showed SA possesses the predominant O-H stretching intense peak at 3253 cm^−1^ and medium intensity peaks at 1569, 1405, and 1025 cm^−1^, which is similar to this study, which supports the notion of high chemical compatibility of excipients [48]. Thermal capacity (°C) is also displayed in Figure 5, and was measured by means of DSC; a variety of materials can be thermally analyzed using DSC [49,50]. Thermal characteristics of individual excipients and formulations as a whole are displayed, in a similar manner to the chemical analysis previously discussed. SA possesses the greatest thermal capacity, surpassing 170 °C, whereas poloxamer was the least thermally resistant. Overall, all formulations depicted high thermal stability exceeding 150 °C, likely a consequence of the fact that all formulations possessed similar quantities of SA, which had chemically bonded to other excipients as previously discussed—thereby shifting thermal resistance of formulations globally. Such stability is consistent with our published studies and suggest long-term performance and appropriate bio-compatibility, consistent with previously published data [48,51,52,53].

## 4. Conclusions

This study has highlighted the favorable outcomes derived from encapsulation of pancreatic islet cells with a BA-Eudragit NM30D^®^ coating in the scope of live tissue transplant for the treatment of diabetes. Improvements in cellular viability, biological activities, inflammatory, and insulin profiles of an encapsulated mouse-cloned pancreatic β-cell line revealed the extent of therapeutic effects. Chemical and thermal analyses of capsule structure highlighted capsules’ chemical interactions and high thermal stability. Most notably, therapeutic effects varied widely across formulations as BA-Eudragit NM30D^®^ ratio altered. This indicates that subtle shifts in β-cell microenvironment influence biological processes and thus insulin production of (potentially) transplanted cells. These findings supplement the knowledge pool, which should be considered in future studies of pancreatic β-cell transplantation for the treatment of diabetes.

## Figures and Tables

**Table 1:** Primary chemical atomic-bond transmittance (%T) of formulation excipients (top, left) and micro/ nano capsules (top, right) as attained through FTIR.

Primary Chemical Atomic-Bond of % Transmittance (%T) over Wavenumber of Formulations’ Excipients		Primary Chemical Atomic-Bond of % Transmittance (%T) over Wavenumber of Micro/Nano Capsules		
			Peak 1	Peak 2
SA	1043 ± 20	F1	1342 ± 10	956 ± 5
CDCA	17,012 ± 200	F2	1406 ± 15	956 ± 20
NM30D^®^	333 ± 10	F3	1640 ± 20	1022 ± 20
Poloxamer	1099 ± 20	F4	1342 ± 50	1101 ± 40
PVP	1096 ± 10	F5	1089 ± 40	1027 ± 40
PLL	1405 ± 30	F6	1075 ± 50	1701 ± 40
		F7	1726 ± 50	1156 ± 30
		F8	1727 ± 40	1167 ± 35

SA = sodium alginate; CDCA = chenodeoxycholic acid; Eudragit NM30D^®^; PVP = Polyvinylpyrrolidone; PLL = Poly-L-Lysine.). Data are mean ± standard error of the mean, *n* = 3.

## Data Availability

The data presented in this study are available on request from the corresponding author. The data are not publicly available due to author property agreements.
